# Crystal structure of SrCo_4_(OH)(PO_4_)_3_, a new hy­droxy­phosphate

**DOI:** 10.1107/S2056989020007331

**Published:** 2020-06-05

**Authors:** Fatima-Zahra Cherif, Mhamed Taibi, Ali Boukhari, Jilali Aride, Abderrazzak Assani, Mohamed Saadi, Lahcen El Ammari

**Affiliations:** a Laboratoire de Chimie Appliquée des Matériaux, Centre des Sciences des Matériaux, Faculty of Science, Mohammed V University in Rabat, Avenue Ibn Batouta, BP 1014, Rabat, Morocco; b Laboratoire de Physico-Chimie des Matériaux Inorganiques et Organiques, Centre des Sciences des Matériaux, Ecole Normale Supérieure, Mohammed V University in Rabat, Morocco

**Keywords:** crystal structure, orthophosphate, cobalt, strontium, bifurcated hydrogen bonding, infrared spectroscopy

## Abstract

Single crystals of strontium tetra­cobalt tris­(orthophosphate) hydroxide, SrCo_4_(OH)(PO_4_)_3_, were grown serendipitously under hydro­thermal conditions at 473 K. The crystal structure consists of undulating chains of edge-sharing [CoO_6_] octa­hedra that are linked into (010) layers by common vertices between chains. Adjacent layers are linked along [010] into a framework structure by tetra­hedral [CoO_4_] units and by PO_4_ tetra­hedra. The framework delimits channels extending along [100] in which the eleven-coordinate strontium cations are situated. Bifurcated O—H⋯O hydrogen bonds of weak strengths consolidate the crystal packing. The title compound was also characterized by infrared spectroscopy.

## Abstract 2

Three [CoO_6_] octa­hedra, one [CoO_4_] tetra­hedron and three PO_4_ tetra­hedra are linked into a three-dimensional framework structure exhibiting channels parallel to [100] in which the eleven-coordinate strontium cations are located.

## Chemical context   

The search for new inorganic materials with open-frame structures comprising transition-metal polyhedra, [*M*O_*x*_], with tetra­hedral phosphate or vanadate units by sharing corners or edges is still ongoing (Rghioui *et al.*, 2019 ▸; Ouaatta *et al.*, 2019 ▸; Khmiyas *et al.*, 2020 ▸). Generally, these inter­connections can lead to structures with cages, inter­layer spaces or channels, and the corresponding compounds are explored extensively for their excellent physical properties and various applications in electrical, electrochemical, magnetic or catalytic processes (Goodenough *et al.*, 1976 ▸; Borel *et al.*, 1991 ▸; La Parola *et al.*, 2018 ▸; Hadouchi *et al.*, 2019 ▸). The introduction of borate groups (BO_3_ or BO_4_) to phosphate (PO_4_) units leads to a group of borophosphates with specific structural characteristics. Compounds of this family likewise exhibit remarkable physicochemical properties that allow them to be applied in different fields (Kniep *et al.*, 1998 ▸; Ewald *et al.*, 2007 ▸; Lin *et al.*, 2008 ▸; Menezes *et al.*, 2008 ▸). About a decade ago, we managed to synthesize two borophosphate phases, *viz*. (Ag_0.57_Ni_0.22_)Ni(H_2_O)_2_[BP_2_O_8_]·0.67H_2_O and AgMg(H_2_O)_2_[BP_2_O_8_]·H_2_O (Zouihri *et al.*, 2011*a* ▸,*b* ▸). In this context, we attempted to synthesize a strontium- and cobalt-based borophosphate, namely SrCo_2_BPO_7_, by means of the hydro­thermal process. Instead, we have isolated a new hy­droxy­phosphate, SrCo_4_(OH)(PO_4_)_3_, and report here its crystal structure and its infrared spectrum.

## Structural commentary   

**Figure 1:**
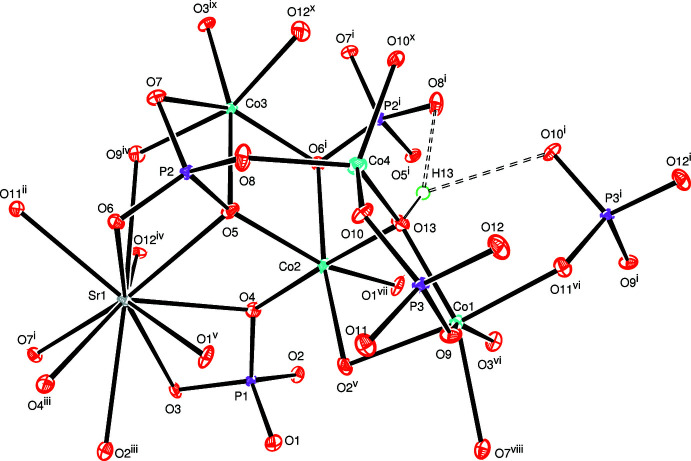
The inter­connection of [CoO_6_] and [CoO_4_] polyhedra, and the hydroxide group linked to PO_4_ tetra­hedra through a bifurcated hydrogen bond (dashed lines). Displacement ellipsoids are drawn at the 50% probability level. [Symmetry codes: (i) −*x*, *y* + 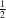, −*z* + 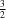; (ii) −*x* − 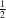, −*y* + 1, *z* + 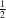; (iii) *x* − 1, *y*, *z*; (iv) −*x* + 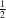, −*y* + 1, *z* + 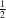; (v) *x* − 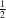, −*y* + 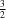, −*z* + 1; (vi) *x* + 1, *y*, *z*; (vii) *x* + 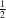, −*y* + 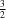, −*z* + 1; (viii) −*x* + 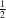, −*y* + 1, *z* − 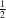; (ix) −*x* + 1, *y* − 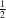, −*z* + 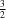; (*x*) *x* + 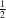, −*y* + 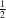, −*z* + 1].

In the three-dimensional framework structure of SrCo_4_(OH)(PO_4_)_3_, an octa­hedral coordination of three cobalt atoms (Co1, Co2, Co3) and a tetra­hedral coordination of the fourth cobalt (Co4) is observed. Atom O13 bears a hydrogen atom and bridges two of the six-coordinate Co atoms (Co1, Co2) and the four-coordinate Co4 atom. The hydroxide group also forms a weak bifurcated hydrogen bond (Table 1 ▸) to two phosphate tetra­hedra (Fig. 1 ▸).

**Figure 2:**
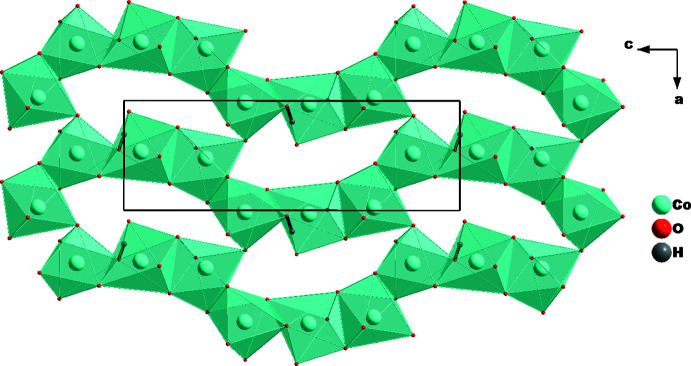
[CoO_6_] octa­hedra sharing edges to form chains that are linked together *via* a common corner every three octa­hedra.

**Figure 3:**
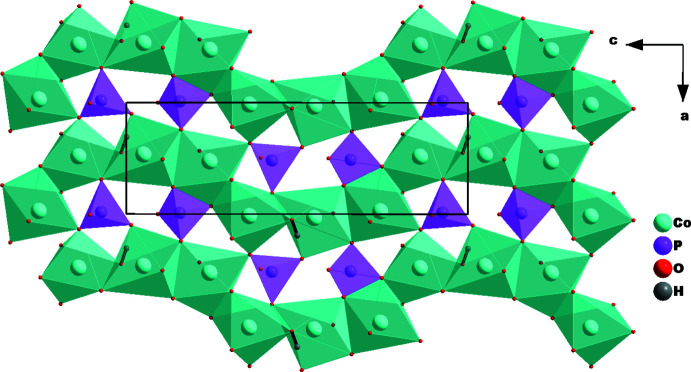
Edge-sharing [CoO_6_] octa­hedra and PO_4_ tetra­hedra building a layer parallel to (010).

The [CoO_6_] octa­hedra share edges to form infinite undulating chains extending parallel to [001]. Adjacent chains are cross-linked *via* common vertex atoms (O3) to build up (010) layers (Fig. 2 ▸) with the formation of oval voids surrounded by eight octa­hedra. Two PO_4_ tetra­hedra occupy the void space, whereby P1O_4_ shares three of its vertices with five [CoO_6_] octa­hedra and P2O_4_ shares an edge with an octa­hedron and a vertex with two opposite octa­hedra (Fig. 3 ▸).

**Figure 4:**
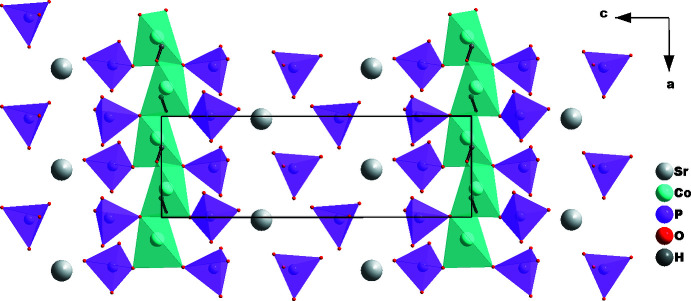
Ribbons formed by [CoO_4_] and P2O_4_ and P3O_4_ tetra­hedra, and strontium atoms and P1O_4_ tetra­hedra at the same height forming the second layer parallel to (010).

**Figure 5:**
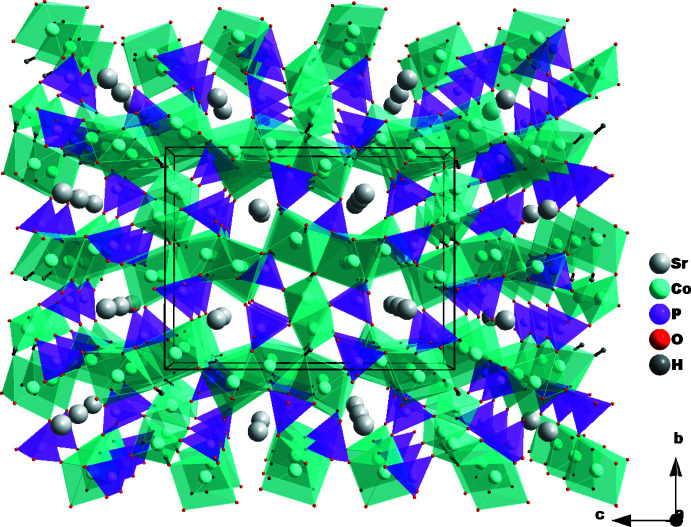
The crystal structure of SrCo_4_(OH)(PO_4_)_3_ in a projection along [100], showing channels in which the strontium cations are located.

The [Co4O_3_OH] tetra­hedra are linked through corners into zigzag chains running parallel to [100]; the chains are flanked by P2O_4_ and P3O_4_ tetra­hedra into ribbons. The strontium cations and P1O_4_ tetra­hedra are of the same height as the ribbons, thus defining a second layer parallel to (010) (Fig. 4 ▸). The crystal structure can be described by the stacking of the two types of layers along [010], which leads to the formation of channels extending parallel to [100] in which the strontium cations are located (Fig. 5 ▸). Each Sr^II^ atom is surrounded by eleven oxygen atoms, forming a distorted polyhedron.

Comparison of the metal–oxygen polyhedra in the title structure with the same type of polyhedra in comparable structures shows a similar behaviour. All [CoO_6_] octa­hedra in SrCo_4_(OH)(PO_4_)_3_ are distorted, with the Co—O distance varying between 2.022 (2) and 2.284 (2) Å. The averaged Co—O distances of 2.130 Å for Co1, 2.122 Å for Co2 and 2.124 Å for Co3 are in good agreement with those of Co_5_(PO_4_)_2_(OH)_4_ (average Co—O distances are 2.107 Å for Co1, 2.144 Å for Co2, 2.140 Å for Co3, 2.148 Å for Co4 and 2.150 Å for Co5; Ruszala *et al.*, 1977 ▸). The distorted [Co4O_3_OH] tetra­hedron shows much shorter Co—O distances ranging from 1.942 (2) to 1.995 (2) Å. These distances are comparable with the averaged ^[4]^Co—O distances of 1.966, 1.955, 1.957 and 1.958 Å observed, respectively, in the phosphates NaCoPO_4_, KCoPO_4_, NH_4_CoPO_4_-Hex and NH_4_CoPO_4_-ABW (Feng *et al.*, 1997 ▸). The PO_4_ tetra­hedra in the title structure have averaged distances of 1.542 Å for P1, 1.539 Å for P2 and 1.539Å for P3, and are compatible with the P—O distances in the orthophosphate SrCo_2_Fe(PO_4_)_3_ (Bouraima *et al.*, 2016 ▸).

The structure model of SrCo_4_(OH)(PO_4_)_3_ is in good agreement with calculations of the bond-valence sums (Brown & Altermatt, 1985 ▸). The obtained values (in valence units) for the cations Sr^II^, Co^II^, and P^V^ are close to the expected values: Sr1 (1.95), Co1 (1.89), Co2 (1.89), Co3 (1.88), Co4 (1.92), P1 (4.90), P2 (4.95) and P3 (4.95). The bond-valence sums calculated for the oxygen atoms range between 1.82 and 2.29 valence units.

## Infrared spectroscopy   

**Figure 6:**
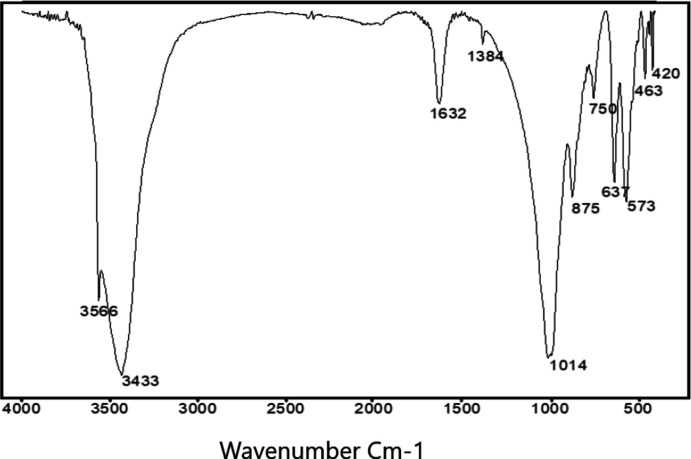
Infrared spectrum of SrCo_4_(OH)(PO_4_)_3_.

An infrared spectrum of SrCo_4_(OH)(PO_4_)_3_ was recorded in order to verify the existence of the hydroxyl and PO_4_ groups in the title compound (Fig. 6 ▸). The FT-IR spectrum shows characteristic vibration bands of isolated PO_4_ groups. The bands observed at around 420 and 463 cm^−1^ can be assigned to the *ν*_2_ asymmetric stretching mode while the vibration at 573 cm^−1^ is attributed to *ν*_4_ asymmetric O—P—O deformation. The weak band observed at 750 cm^−1^ most likely originates from ^[4]^Co—O vibrations, as observed in many other phosphates (Rusakov *et al.*, 2006 ▸; Antony *et al.*, 2011 ▸; Bushiri *et al.*, 2002 ▸; De Pedro *et al.*, 2010 ▸). The vibration at 1014 cm^−1^ corresponds to the *ν*_3_ asymmetric stretching mode of the phosphate tetra­hedra. The remaining vibrations centred at 3566, 3433 and 1632 cm^−1^ are commonly assigned to the stretching vibration of the bridging –OH group, as in Co_2_PO_4_OH (Wang *et al.*, 2014 ▸), in addition to the OH^−^ librational mode, which is observed at 637 cm^−1^. We also note the presence of bands at 1384 and 875 cm^−1^, indicating C—O bonds (Ribeiro *et al.*, 2006 ▸). This observation suggests that the powdered sample contained impurities of a carbonate. The assignments of all vibration bands are summarized in Table 2 ▸.

## Database survey   

A search in the Inorganic Crystal Structure Database (ICSD; Zagorac *et al.*, 2019 ▸) revealed no match in the pseudo-quaternary system SrO/CoO/P_2_O_5_/OH. However, five compounds were identified in the pseudo-ternary SrO/CoO/P_2_O_5_ system, *viz*. triclinic SrCo_2_(PO_4_)_2_ (*P* 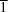, *Z* = 2; El Bali *et al.*, 1993 ▸), monoclinic SrCoP_2_O_7_ (*P*2_1_/*n*, *Z* = 4; Riou & Raveau 1991 ▸), monoclinic Sr_2_Co(PO_4_)_2_ and SrCo_3_(P_2_O_7_)_2_ (both *P*2_1_/*c*, *Z* = 6 and 2, respectively; Belik *et al.* 2001 ▸ and Yang *et al.*, 2008 ▸) and hexa­gonal Sr_5_Co_0.18_P_3_O_12.92_ (*P*6_3_/*m*, *Z* = 2; Kazin *et al.*, 2017 ▸).

## Synthesis and crystallization   

Single crystals of SrCo_4_(OH)(PO_4_)_3_ were obtained serendi­pitously by attempting to synthesize the borophosphate SrCo_2_BPO_7_ under hydro­thermal conditions. The starting materials, Sr(NO_3_)_2_ (0.3174 g), Co(CH_3_COO)_2_·4H_2_O (0.7473 g), H_3_BO_3_ (0.0927 g) and H_3_PO_4_ (12 N; 0.1 ml), were mixed in the molar proportions of 1:2:1:1. The hydro­thermal reaction was conducted in a 23 ml Teflon-lined autoclave, filled to 50% with distilled water and heated under autogenous pressure at 473 K for five days. After the end of the heat treatment, the autoclave was taken out of the oven and allowed to cool to room temperature. The reaction product was collected, filtered, rinsed with distilled water and dried in air. Optical microscopy revealed two types of crystals, *viz*. dark-purple and dark-red rectangular crystals. X-ray diffraction analysis showed the red crystals to be Co_2_(OH)PO_4_ (Harrison *et al.*, 1995 ▸). The purple parallelepipeds correspond to the title compound.

Infrared spectroscopic measurements were performed on a VERTEX 70 FT-IR spectrometer, using the MRI transmission technique using KBr pellets. An adequate qu­antity of the studied phosphate powder, obtained by grinding the SrCo_4_(OH)(PO_4_)_3_ crystals, was diluted in KBr before being pressed into a pellet. The analysis was performed at room temperature, and the spectrum was recorded in the range 4000–400 cm^−1^.

## Refinement   

Crystal data, data collection and structure refinement details are summarized in Table 3 ▸. The hydrogen atom of the OH group was located in a difference-Fourier map and was refined was a fixed O—H bond length of 0.82 Å and *U*_iso_(H) = 1.5*U*_eq_(O). The maximum and minimum remaining electron density was located at 0.69 Å from Sr1 and 0.56 Å from Co4, respectively. The reflection (011) was affected by the beam-stop (*F*_o_^2^ = 0) while reflections (052) and (053), having *F*_o_^2^ > *F*_c_^2^, were probably affected by the Renninger effect. All three reflections were omitted from the refinement.

## Supplementary Material

Crystal structure: contains datablock(s) I. DOI: 10.1107/S2056989020007331/wm5562sup1.cif

Structure factors: contains datablock(s) I. DOI: 10.1107/S2056989020007331/wm5562Isup2.hkl

CCDC reference: 2006988

Additional supporting information:  crystallographic information; 3D view; checkCIF report

## Figures and Tables

**Table 1:** Hydrogen-bond geometry (Å, °)

*D*—H⋯*A*	*D*—H	H⋯*A*	*D*⋯*A*	*D*—H⋯*A*
O13—H13⋯O8^i^	0.82	2.21	2.982 (3)	157
O13—H13⋯O10^i^	0.82	2.40	3.010 (3)	132

Symmetry code: (i) 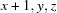.

**Table 2:** Assignments of infrared vibration bands (cm^−1^) for SrCo_4_(OH)(PO_4_)_3_

Position	Assignment
420	ν_2_ asymmetric stretching mode of P—O bonds
463	ν_2_ asymmetric stretching mode of P—O bond
573	ν_4_ asymmetric deformation of O—P—O
637	ν_L_ libration mode of the hydroxyl group
750	^[4]^Co—O stretching mode
875	ν_2_ vibration of C—O bond
1014	ν_3_ asymmetric stretching mode of PO_4_^3−^
1384	ν_3_ vibration of C—O bond
1632	O—H stretching band
3433	O—H stretching band
3566	ν_s_ stretching mode of the hydroxyl group^−^

**Table 3:** Experimental details

Crystal data	
Chemical formula	SrCo_4_(OH)(PO_4_)_3_
*M*_r_	625.26
Crystal system, space group	Orthorhombic, *P*2_1_2_1_2_1_
Temperature (K)	296
*a*, *b*, *c* (Å)	5.1245 (1), 12.0491 (2), 15.7118 (3)
*V* (Å^3^)	970.13 (3)
*Z*	4
Radiation type	Mo *K*α
μ (mm^−1^)	12.74
Crystal size (mm)	0.35 × 0.26 × 0.17

Data collection	
Diffractometer	Bruker X8 APEX
Absorption correction	Multi-scan (*SADABS*; Krause *et al.*, 2015 ▸)
*T*_min_, *T*_max_	0.391, 0.748
No. of measured, independent and observed [*I* > 2σ(*I*)] reflections	34471, 4243, 4038
*R*_int_	0.045
(sin θ/λ)_max_ (Å^−1^)	0.806

Refinement	
*R*[*F* ^2^ > 2σ(*F* ^2^)], *wR*(*F* ^2^), *S*	0.022, 0.050, 1.07
No. of reflections	4243
No. of parameters	191
H-atom treatment	H-atom parameters constrained
Δρ_max_, Δρ_min_ (e Å^−3^)	1.60, −0.68
Absolute structure	Flack *x* determined using 1625 quotients [(*I* ^+^)-(*I* ^-^)]/[(*I* ^+^)+(*I* ^-^)] (Parsons *et al.*, 2013 ▸).
Absolute structure parameter	0.008 (3)

Computer programs: *APEX2* and *SAINT* (Bruker, 2016 ▸), *SHELXT2014/5* (Sheldrick, 2015*a* ▸), *SHELXL2018/3* (Sheldrick, 2015*b* ▸), *ORTEP-3 for Windows* (Farrugia, 2012 ▸), *DIAMOND* (Brandenburg, 2006 ▸) and *publCIF* (Westrip, 2010 ▸).

## supplementary crystallographic information

### Crystal data

**Table:**

SrCo_4_(OH)(PO_4_)_3_	*D*_x_ = 4.281 Mg m^−^^3^
*M**_r_* = 625.26	Mo *K*α radiation, λ = 0.71073 Å
Orthorhombic, *P*2_1_2_1_2_1_	Cell parameters from 4243 reflections
*a* = 5.1245 (1) Å	θ = 2.6–35.0°
*b* = 12.0491 (2) Å	µ = 12.74 mm^−^^1^
*c* = 15.7118 (3) Å	*T* = 296 K
*V* = 970.13 (3) Å^3^	Parallelepiped, violet
*Z* = 4	0.35 × 0.26 × 0.17 mm
*F*(000) = 1184	

### Data collection

**Table:**

Bruker X8 APEX Diffractometer	4243 independent reflections
Radiation source: fine-focus sealed tube	4038 reflections with *I* > 2σ(*I*)
Graphite monochromator	*R*_int_ = 0.045
φ and ω scans	θ_max_ = 35.0°, θ_min_ = 2.6°
Absorption correction: multi-scan (SADABS; Krause *et al.*, 2015)	*h* = −8→8
*T*_min_ = 0.391, *T*_max_ = 0.748	*k* = −19→18
34471 measured reflections	*l* = −25→25

### Refinement

**Table:**

Refinement on *F*^2^	H-atom parameters constrained
Least-squares matrix: full	*w* = 1/[σ^2^(*F*_o_^2^) + (0.0221*P*)^2^] where *P* = (*F*_o_^2^ + 2*F*_c_^2^)/3
*R*[*F*^2^ > 2σ(*F*^2^)] = 0.022	(Δ/σ)_max_ = 0.001
*wR*(*F*^2^) = 0.050	Δρ_max_ = 1.60 e Å^−^^3^
*S* = 1.07	Δρ_min_ = −0.68 e Å^−^^3^
4243 reflections	Extinction correction: SHELXL-2018/3 (Sheldrick, 2015*b*), Fc^*^=kFc[1+0.001xFc^2^λ^3^/sin(2θ)]^-1/4^
191 parameters	Extinction coefficient: 0.0042 (3)
0 restraints	Absolute structure: Flack *x* determined using 1625 quotients [(*I*^+^)-(*I*^-^)]/[(*I*^+^)+(*I*^-^)] (Parsons *et al.*, 2013).
Hydrogen site location: difference Fourier map	Absolute structure parameter: 0.008 (3)

### Special details

**Table:**

Geometry. All esds (except the esd in the dihedral angle between two l.s. planes) are estimated using the full covariance matrix. The cell esds are taken into account individually in the estimation of esds in distances, angles and torsion angles; correlations between esds in cell parameters are only used when they are defined by crystal symmetry. An approximate (isotropic) treatment of cell esds is used for estimating esds involving l.s. planes.

### Fractional atomic coordinates and isotropic or equivalent isotropic displacement parameters (Å^2^)

**Table:**

	*x*	*y*	*z*	*U*_iso_*/*U*_eq_	
Sr1	0.02677 (6)	0.72309 (2)	0.67782 (2)	0.00840 (6)	
Co1	0.51704 (8)	0.48429 (3)	0.35972 (3)	0.00572 (8)	
Co2	0.53401 (9)	0.58186 (4)	0.54997 (3)	0.00613 (8)	
Co3	0.47299 (8)	0.46200 (3)	0.74367 (3)	0.00580 (8)	
Co4	0.28203 (8)	0.32358 (4)	0.51444 (3)	0.00794 (9)	
P1	0.51747 (15)	0.85171 (6)	0.57349 (5)	0.00425 (12)	
P2	0.01127 (15)	0.45034 (6)	0.65719 (5)	0.00471 (13)	
P3	−0.00586 (15)	0.30856 (6)	0.33173 (5)	0.00413 (12)	
O1	0.4003 (4)	0.8648 (2)	0.48533 (15)	0.0092 (4)	
O2	0.8108 (4)	0.8848 (2)	0.57323 (15)	0.0074 (4)	
O3	0.3638 (4)	0.9243 (2)	0.63798 (15)	0.0074 (4)	
O4	0.4924 (5)	0.73162 (18)	0.60637 (15)	0.0095 (4)	
O5	0.2427 (4)	0.5287 (2)	0.63664 (15)	0.0078 (4)	
O6	−0.2433 (4)	0.5173 (2)	0.66027 (15)	0.0075 (4)	
O7	0.0710 (4)	0.40910 (19)	0.74879 (15)	0.0068 (4)	
O8	−0.0011 (5)	0.3572 (2)	0.59242 (14)	0.0108 (4)	
O9	0.1926 (4)	0.40027 (19)	0.30856 (15)	0.0073 (4)	
O10	0.0074 (4)	0.29074 (18)	0.43053 (14)	0.0080 (4)	
O11	−0.2813 (4)	0.34923 (19)	0.30965 (15)	0.0075 (4)	
O12	0.0671 (4)	0.20305 (19)	0.28586 (16)	0.0097 (4)	
O13	0.5519 (4)	0.43152 (18)	0.48508 (15)	0.0073 (4)	
H13	0.691669	0.405651	0.500919	0.011*	

### Atomic displacement parameters (Å^2^)

**Table:**

	*U*^11^	*U*^22^	*U*^33^	*U*^12^	*U*^13^	*U*^23^
Sr1	0.01002 (12)	0.00611 (12)	0.00908 (12)	0.00095 (10)	0.00229 (10)	0.00061 (10)
Co1	0.00609 (16)	0.00557 (17)	0.00550 (17)	−0.00054 (14)	−0.00005 (14)	−0.00035 (13)
Co2	0.00739 (17)	0.00508 (17)	0.00593 (17)	−0.00087 (15)	0.00149 (14)	−0.00104 (13)
Co3	0.00560 (16)	0.00635 (17)	0.00544 (16)	0.00038 (14)	0.00019 (14)	0.00037 (13)
Co4	0.00687 (17)	0.0095 (2)	0.00748 (18)	−0.00059 (14)	−0.00076 (14)	0.00135 (16)
P1	0.0047 (3)	0.0041 (3)	0.0039 (3)	−0.0005 (3)	0.0001 (2)	0.0001 (2)
P2	0.0044 (3)	0.0055 (3)	0.0042 (3)	0.0001 (3)	−0.0001 (2)	0.0002 (2)
P3	0.0051 (3)	0.0035 (3)	0.0038 (3)	−0.0002 (2)	−0.0002 (2)	0.0000 (2)
O1	0.0093 (9)	0.0138 (11)	0.0045 (9)	0.0012 (8)	−0.0026 (8)	−0.0010 (9)
O2	0.0044 (9)	0.0098 (10)	0.0079 (10)	−0.0026 (8)	0.0002 (7)	−0.0008 (8)
O3	0.0069 (9)	0.0083 (10)	0.0071 (10)	0.0033 (8)	−0.0001 (7)	−0.0022 (9)
O4	0.0140 (10)	0.0036 (9)	0.0109 (9)	−0.0001 (9)	0.0049 (8)	0.0008 (7)
O5	0.0058 (9)	0.0099 (10)	0.0077 (10)	−0.0020 (8)	0.0004 (7)	0.0028 (9)
O6	0.0061 (9)	0.0099 (11)	0.0065 (10)	0.0027 (8)	−0.0002 (7)	0.0016 (8)
O7	0.0069 (9)	0.0073 (10)	0.0062 (9)	−0.0016 (7)	−0.0012 (7)	0.0015 (8)
O8	0.0115 (10)	0.0114 (10)	0.0094 (10)	−0.0022 (9)	0.0030 (9)	−0.0059 (8)
O9	0.0077 (9)	0.0055 (9)	0.0088 (10)	−0.0020 (7)	0.0004 (7)	0.0022 (8)
O10	0.0095 (10)	0.0104 (10)	0.0039 (9)	−0.0020 (8)	0.0000 (8)	0.0008 (7)
O11	0.0062 (9)	0.0081 (10)	0.0082 (10)	0.0008 (8)	−0.0016 (7)	−0.0017 (8)
O12	0.0120 (10)	0.0060 (10)	0.0111 (10)	0.0014 (8)	−0.0002 (8)	−0.0035 (8)
O13	0.0068 (9)	0.0058 (9)	0.0092 (9)	0.0006 (7)	−0.0002 (8)	0.0003 (8)

### Geometric parameters (Å, º)

**Table:**

Sr1—O7^i^	2.570 (2)	Co3—O6^vi^	2.067 (2)
Sr1—O11^ii^	2.575 (2)	Co3—O3^ix^	2.089 (2)
Sr1—O4	2.639 (2)	Co3—O12^x^	2.098 (2)
Sr1—O5	2.669 (2)	Co3—O9^iv^	2.125 (2)
Sr1—O2^iii^	2.779 (2)	Co3—O7	2.158 (2)
Sr1—O12^iv^	2.829 (2)	Co3—O5	2.206 (2)
Sr1—O1^v^	2.848 (2)	Co4—O8	1.942 (2)
Sr1—O6	2.853 (2)	Co4—O13	1.954 (2)
Sr1—O9^iv^	2.915 (2)	Co4—O10	1.969 (2)
Sr1—O4^iii^	2.961 (2)	Co4—O10^x^	1.995 (2)
Sr1—O3	3.041 (2)	P1—O1	1.518 (2)
Co1—O13	2.077 (2)	P1—O4	1.542 (2)
Co1—O11^vi^	2.082 (2)	P1—O3	1.553 (2)
Co1—O3^vii^	2.091 (2)	P1—O2	1.555 (2)
Co1—O9	2.106 (2)	P2—O8	1.517 (2)
Co1—O2^v^	2.171 (2)	P2—O6	1.534 (2)
Co1—O7^viii^	2.212 (2)	P2—O5	1.550 (2)
Co2—O4	2.022 (2)	P2—O7	1.553 (2)
Co2—O1^vii^	2.060 (2)	P3—O12	1.508 (2)
Co2—O13	2.081 (2)	P3—O11	1.534 (2)
Co2—O5	2.120 (2)	P3—O9	1.545 (2)
Co2—O6^vi^	2.216 (2)	P3—O10	1.569 (2)
Co2—O2^v^	2.284 (2)	O13—H13	0.8200
			
O7^i^—Sr1—O11^ii^	80.73 (7)	O9—Co1—O2^v^	98.63 (9)
O7^i^—Sr1—O4	109.46 (7)	O13—Co1—O7^viii^	160.47 (9)
O11^ii^—Sr1—O4	142.85 (7)	O11^vi^—Co1—O7^viii^	104.92 (9)
O7^i^—Sr1—O5	162.67 (7)	O3^vii^—Co1—O7^viii^	83.16 (8)
O11^ii^—Sr1—O5	95.77 (7)	O9—Co1—O7^viii^	79.48 (8)
O4—Sr1—O5	63.66 (7)	O2^v^—Co1—O7^viii^	82.02 (9)
O7^i^—Sr1—O2^iii^	64.92 (7)	O4—Co2—O1^vii^	86.30 (10)
O11^ii^—Sr1—O2^iii^	121.22 (7)	O4—Co2—O13	175.22 (10)
O4—Sr1—O2^iii^	94.66 (7)	O1^vii^—Co2—O13	95.72 (9)
O5—Sr1—O2^iii^	129.56 (7)	O4—Co2—O5	85.04 (9)
O7^i^—Sr1—O12^iv^	66.42 (7)	O1^vii^—Co2—O5	155.36 (9)
O11^ii^—Sr1—O12^iv^	89.04 (7)	O13—Co2—O5	94.75 (9)
O4—Sr1—O12^iv^	65.03 (7)	O4—Co2—O6^vi^	91.44 (10)
O5—Sr1—O12^iv^	96.69 (7)	O1^vii^—Co2—O6^vi^	81.43 (9)
O2^iii^—Sr1—O12^iv^	115.29 (7)	O13—Co2—O6^vi^	93.14 (9)
O7^i^—Sr1—O1^v^	133.13 (7)	O5—Co2—O6^vi^	75.77 (9)
O11^ii^—Sr1—O1^v^	119.13 (7)	O4—Co2—O2^v^	99.31 (9)
O4—Sr1—O1^v^	80.64 (7)	O1^vii^—Co2—O2^v^	99.98 (9)
O5—Sr1—O1^v^	63.27 (7)	O13—Co2—O2^v^	76.10 (9)
O2^iii^—Sr1—O1^v^	68.76 (7)	O5—Co2—O2^v^	104.17 (9)
O12^iv^—Sr1—O1^v^	145.52 (7)	O6^vi^—Co2—O2^v^	169.22 (9)
O7^i^—Sr1—O6	134.98 (7)	O6^vi^—Co3—O3^ix^	110.65 (9)
O11^ii^—Sr1—O6	63.04 (7)	O6^vi^—Co3—O12^x^	90.20 (10)
O4—Sr1—O6	115.55 (7)	O3^ix^—Co3—O12^x^	84.17 (10)
O5—Sr1—O6	54.21 (6)	O6^vi^—Co3—O9^iv^	109.48 (9)
O2^iii^—Sr1—O6	111.05 (7)	O3^ix^—Co3—O9^iv^	84.42 (9)
O12^iv^—Sr1—O6	133.49 (7)	O12^x^—Co3—O9^iv^	159.76 (10)
O1^v^—Sr1—O6	58.64 (7)	O6^vi^—Co3—O7	142.20 (9)
O7^i^—Sr1—O9^iv^	102.99 (7)	O3^ix^—Co3—O7	106.54 (9)
O11^ii^—Sr1—O9^iv^	60.11 (7)	O12^x^—Co3—O7	87.01 (9)
O4—Sr1—O9^iv^	82.74 (7)	O9^iv^—Co3—O7	80.30 (9)
O5—Sr1—O9^iv^	61.25 (7)	O6^vi^—Co3—O5	77.04 (9)
O2^iii^—Sr1—O9^iv^	166.11 (7)	O3^ix^—Co3—O5	166.41 (9)
O12^iv^—Sr1—O9^iv^	51.30 (6)	O12^x^—Co3—O5	107.46 (10)
O1^v^—Sr1—O9^iv^	123.85 (7)	O9^iv^—Co3—O5	82.38 (9)
O6—Sr1—O9^iv^	82.19 (6)	O7—Co3—O5	68.00 (8)
O7^i^—Sr1—O4^iii^	87.68 (6)	O8—Co4—O13	122.63 (10)
O11^ii^—Sr1—O4^iii^	82.23 (7)	O8—Co4—O10	86.00 (10)
O4—Sr1—O4^iii^	132.33 (9)	O13—Co4—O10	118.80 (9)
O5—Sr1—O4^iii^	108.78 (7)	O8—Co4—O10^x^	107.64 (10)
O2^iii^—Sr1—O4^iii^	51.92 (6)	O13—Co4—O10^x^	98.76 (9)
O12^iv^—Sr1—O4^iii^	153.73 (7)	O10—Co4—O10^x^	124.43 (5)
O1^v^—Sr1—O4^iii^	57.40 (6)	O1—P1—O4	111.72 (14)
O6—Sr1—O4^iii^	62.94 (6)	O1—P1—O3	109.66 (13)
O9^iv^—Sr1—O4^iii^	137.82 (6)	O4—P1—O3	105.51 (13)
O7^i^—Sr1—O3	60.53 (7)	O1—P1—O2	110.70 (14)
O11^ii^—Sr1—O3	135.45 (7)	O4—P1—O2	108.80 (14)
O4—Sr1—O3	50.80 (6)	O3—P1—O2	110.33 (13)
O5—Sr1—O3	114.45 (6)	O8—P2—O6	112.00 (14)
O2^iii^—Sr1—O3	62.97 (6)	O8—P2—O5	110.08 (14)
O12^iv^—Sr1—O3	56.98 (7)	O6—P2—O5	109.67 (13)
O1^v^—Sr1—O3	103.90 (6)	O8—P2—O7	113.16 (14)
O6—Sr1—O3	161.48 (6)	O6—P2—O7	107.85 (13)
O9^iv^—Sr1—O3	105.74 (6)	O5—P2—O7	103.72 (12)
O4^iii^—Sr1—O3	114.80 (6)	O12—P3—O11	112.92 (13)
O13—Co1—O11^vi^	94.38 (9)	O12—P3—O9	109.08 (14)
O13—Co1—O3^vii^	94.12 (9)	O11—P3—O9	108.90 (13)
O11^vi^—Co1—O3^vii^	89.80 (9)	O12—P3—O10	110.29 (14)
O13—Co1—O9	106.41 (9)	O11—P3—O10	107.90 (13)
O11^vi^—Co1—O9	82.65 (9)	O9—P3—O10	107.61 (13)
O3^vii^—Co1—O9	158.54 (9)	Co4—O13—H13	107.1
O13—Co1—O2^v^	78.70 (9)	Co1—O13—H13	118.6
O11^vi^—Co1—O2^v^	173.06 (9)	Co2—O13—H13	102.7
O3^vii^—Co1—O2^v^	91.29 (9)		

Symmetry codes: (i) −*x*, *y*+1/2, −*z*+3/2; (ii) −*x*−1/2, −*y*+1, *z*+1/2; (iii) *x*−1, *y*, *z*; (iv) −*x*+1/2, −*y*+1, *z*+1/2; (v) *x*−1/2, −*y*+3/2, −*z*+1; (vi) *x*+1, *y*, *z*; (vii) *x*+1/2, −*y*+3/2, −*z*+1; (viii) −*x*+1/2, −*y*+1, *z*−1/2; (ix) −*x*+1, *y*−1/2, −*z*+3/2; (x) *x*+1/2, −*y*+1/2, −*z*+1.

### Hydrogen-bond geometry (Å, º)

**Table:**

*D*—H···*A*	*D*—H	H···*A*	*D*···*A*	*D*—H···*A*
O13—H13···O8^vi^	0.82	2.21	2.982 (3)	157
O13—H13···O10^vi^	0.82	2.40	3.010 (3)	132

Symmetry code: (vi) *x*+1, *y*, *z*.
